# Amniotic Mesenchymal Stromal Cell Administration Prevents and Stops Lung Fibrosis and Is Associated with Distinct Macrophage Signatures

**DOI:** 10.3390/pharmaceutics18081039

**Published:** 2026-08-20

**Authors:** Anna Cargnoni, Serafina Farigu, Pietro Romele, Andrea Papait, Marta Magatti, Antonietta Silini, Ornella Parolini

**Affiliations:** 1 Centro di Ricerca E. Menni, Fondazione Poliambulanza Istituto Ospedaliero, 25124 Brescia, Italy; serafina.farigu@poliambulanza.it (S.F.); pietro.romele@poliambulanza.it (P.R.); marta.magatti@poliambulanza.it (M.M.); antonietta.silini@poliambulanza.it (A.S.); 2 Department of Life Science and Public Health, Università Cattolica del Sacro Cuore, 00168 Rome, Italy; andrea.papait@unicatt.it (A.P.); ornella.parolini@unicatt.it (O.P.); 3 Fondazione Policlinico Universitario “Agostino Gemelli”, Istituto di Ricovero e Cura a Carattere Scientifico (IRCCS), 00168 Rome, Italy; 4 Fondazione IRCCS Casa Sollievo della Sofferenza, 71013 San Giovanni Rotondo, Italy

**Keywords:** amniotic mesenchymal stromal cells, lung macrophages, SPARC^+^ macrophages, CD169^+^ macrophages, lung inflammation, lung fibrosis

## Abstract

**Background/Objectives**: Mesenchymal stromal cells from the amniotic membrane (hAMSCs) counteract fibrosis progression, primarily via anti-inflammatory effects like promoting macrophage polarization toward an anti-inflammatory, pro-regenerative phenotype. **Methods**: We investigated hAMSCs’ ability to prevent and halt lung fibrosis in a bleomycin-induced fibrosis murine model. We focused on their impact on recruitment and polarization of different macrophage populations, including SPARC- and CD169-expressing macrophages, implicated in resolving pulmonary inflammation and fibrosis. **Results**: hAMSCs, administered early (concomitant with bleomycin, during acute inflammation), or late (at day 7 post-bleomycin, during established fibrosis), showed anti-fibrotic activity, preserving alveolar area, reducing the extent of lung fibrosis, and decreasing α-SMA levels. These preventive and late anti-fibrotic effects of hAMSCs are associated with a context-dependent presence of distinct macrophage signatures. Early treatment reduced macrophage recruitment and increased levels of Arg1^+^/iNOS^−^ macrophages, curbing injury-induced inflammation. Late treatment uniquely increased the lung levels of CD169^+^ macrophages, suggesting their contribution to hAMSCs’ anti-fibrotic effect. We hypothesized a potential involvement of lung CD169^+^ macrophages in promoting recruitment of regulatory T cells (Tregs) to the lungs. Although these macrophages can establish a CCL22-CCR4 axis with Tregs, and treatment effectively boosted Treg lung levels, the Treg increase is not directly attributable to higher CD169^+^ macrophage numbers, implying other potentially IL-10-driven mechanisms. **Conclusions**: hAMSC treatment effectively prevents and blocks lung fibrosis. These effects are associated with distinct lung macrophage marker profiles, suggesting a potential involvement of different macrophage populations in a time-dependent manner; thus highlighting administration timing’s role in optimizing therapeutic synergy.

## 1. Introduction

Previous research from our group and others has demonstrated that cells derived from the amniotic membrane, specifically human amniotic mesenchymal stromal cells (hAMSCs) and amniotic epithelial cells, can effectively counteract fibrosis progression in a murine model of bleomycin-induced pulmonary injury [1,2,3]. These protective effects are primarily attributed to their strong anti-inflammatory activity [4], mediated by their secretome, including soluble bioactive factors and extracellular vesicles (EVs). Indeed, in vitro studies have shown that amniotic cells and their secretome can both, equally, regulate immune responses by inhibiting the proliferation of activated T and B lymphocytes [5,6,7]; promoting the polarization of T cells toward a regulatory phenotype (regulatory T cells) [8] and favoring a shift in macrophage polarization from the pro-inflammatory M1 toward the anti-inflammatory M2 phenotype [9].

Notably, these immunomodulatory effects are also evident in vivo. In bleomycin-induced lung fibrosis models, amniotic cells have been shown to reduce total macrophage numbers in lung tissue [1,10,11], increase levels of M2 macrophages [10,12], decrease recruitment of T and B lymphocytes [2] and elevate regulatory T cell populations (Tregs) [2,12]. However, prior studies, including ours, have focused exclusively on the immunomodulatory impact of amniotic cells or their secretome when administered during the early inflammatory phase of bleomycin lung injury, leaving the effects during the established fibrotic phase unexplored. Moreover, investigations into the impact of amniotic cells on lung macrophages have been limited, largely focusing on reduced recruitment and M2 polarization, key anti-fibrotic traits, but overlooking other macrophage populations critical to fibrosis progression or resolution.

Macrophages exhibit diverse roles in lung fibrosis, adopting multiple phenotypes that can either promote fibrogenesis or support inflammation resolution and clearing extracellular matrix (ECM) components [13].

The conventional M1/M2 macrophage classification, based on the expression of markers like inducible nitric oxide synthase (iNOS) for M1, and arginase-1 (Arg1) for M2, oversimplifies macrophage complexity. Distinct macrophage subpopulations indeed contribute variably to lung fibrosis and inflammation [14,15,16], yet a definitive correlation between specific phenotypes and activity remains elusive.

In this context, interstitial lung macrophages clearly play a strong role in maintaining pulmonary homeostasis by regulating both damage- and pathogen-induced inflammation [17,18,19,20].

Based on our previous findings that hAMSCs promote macrophage differentiation toward an anti-inflammatory phenotype in vitro, we hypothesized that their anti-fibrotic effects in vivo might be associated with an enrichment of macrophage populations previously reported to exert pro-resolving and beneficial functions in bleomycin-induced lung fibrosis. In this context, we selected two marker-defined macrophage populations with strong prior links to anti-inflammatory and fibrosis resolution activity: macrophages expressing Secreted Protein Acidic and Rich in Cysteine (SPARC) [21] and those expressing sialoadhesin (CD169), also known as nerve- and airway-associated macrophages (NAMs) [22].

SPARC is a collagen-binding protein, known to affect both collagen ECM assembly and TGF-β activity. Differential cell-type-dependent functions have been suggested for SPARC. In patients with pulmonary fibrosis, SPARC was overexpressed in pulmonary fibroblasts, promoting persistent epithelial activation [23] and prolonged cell survival [24], leading to excess collagen assembly and fibrosis.

Conversely, SPARC depletion from macrophages has been shown to exacerbate inflammation and fibrosis in a model of bleomycin-induced lung fibrosis [21], suggesting that SPARC from macrophages limits fibrosis by reducing the extent of inflammation. Furthermore, alternatively activated macrophages have been shown to control extracellular SPARC concentrations by internalizing it via stabilin-1 for endosomal degradation [23]. Through this mechanism, macrophages can promote the clearance of excess SPARC involved in collagen deposition [25,26], thus actively contributing to fibrosis resolution.

NAMs have been shown to play an important role in regulating lung inflammation in vivo [22], and their depletion has been shown to be linked to increased pro-inflammatory cytokines and monocytic cell infiltration in the lung and broncho-alveolar lavage [22]. Interestingly, NAMs exhibit phenotypic traits (i.e., CD169 expression and IL-10 secretion) that overlap with another anti-fibrotic macrophage subset (Lyve1^hi^MHCII^lo^), which contributes to mitigating bleomycin-induced pulmonary fibrosis [20].

This study evaluates whether hAMSCs can not only prevent but also halt the progression of established lung fibrosis. Furthermore, since macrophages are key regulators of tissue repair and undergo phenotypic and functional changes across the inflammatory, inflammation-resolution and wound-healing phases after tissue injury [27], we hypothesized that distinct macrophage populations contribute to the inflammatory and fibrotic phases of pulmonary disease. In this context, hAMSCs’ anti-fibrotic effects could be associated with the modulation of stage-specific macrophages.

To this end, hAMSCs were administered either simultaneously with bleomycin-induced injury (early treatment, during acute inflammation) or at day 7 post-bleomycin (late treatment, when fibrosis is already established) [28]. This dual-timing approach enabled us to assess the effects of hAMSCs on stage-specific tissue macrophage dynamics during inflammation versus established fibrosis, specifically focusing on SPARC- and CD169-expressing populations.

Our findings demonstrate that hAMSCs possess anti-fibrotic effects regardless of the timing of administration. Notably, this benefit was associated with distinct macrophage marker profiles, suggesting a potential involvement of different macrophage populations that varied based on treatment timing and disease stage.

Early hAMSC treatment significantly decreased macrophage recruitment to the lung and favoured their polarization toward an anti-inflammatory (Arg1 positive) and anti-fibrotic (SPARC negative) phenotype. Conversely, for the first time, we observed that late hAMSC administration correlated with increased levels of CD169-expressing macrophages, suggesting the potential ability of hAMSCs to modulate macrophages in a stage-specific way in bleomycin lung fibrosis.

We hypothesized that CD169^+^ macrophages might exert anti-inflammatory and anti-fibrotic effects through CCL22/CCR4-mediated recruitment of Tregs [29]. Consistent with this, late hAMSC treatment significantly increased Treg levels in the lungs of bleomycin-challenged mice. However, although our data show that CD169^+^ macrophages can establish a CCL22-CCR4 signaling axis with Tregs, our findings still suggest that the rise in Tregs is not directly driven by the increased abundance of CD169^+^ macrophages, indicating that additional mechanisms or cell types may contribute to Treg recruitment in this context.

## 2. Materials and Methods

### 2.1. Ethics Statement

Human term placentae were collected from healthy women after vaginal delivery or caesarean section at term after obtaining written consent, according to the guidelines of the Brescia Provincial Ethics Committee (number NP 2243, 19 January 2016).

Animal experiments were carried out in accordance with the guidelines established by the Italian law DL 26/2014. The experimental protocol was approved by the Italian Ministry of Health (Authorization n. 779/2016-PR) and by the Committee on the Ethics of Animal Experiments of the University of Milan (n. 105/14, 9 December 2014).

### 2.2. hAMSC Isolation and Characterization

A total of 10 amniotic membranes were collected, and mesenchymal stromal cells were isolated as previously described [30]. Membrane fragments (3 × 3 cm^2^) were digested at 37 °C in dispase (2.5 U/mL) (VWR, Radnor, PA, USA) for 9 min and then transferred to RPMI 1640 complete medium (supplemented with 10% heat-inactivated fetal bovine serum, 1% penicillin/streptomycin and 1% L-glutamine) (all from Sigma-Aldrich, St. Louis, MO, USA) to block digestion. The fragments were then incubated in collagenase 0.94 mg/mL and DNase I (both from Roche, Basel, Switzerland) for 2.5–3 h at 37 °C. After centrifugation at low g, the obtained supernatant was filtered through a 100-μm cell strainer (BD Falcon, Bedford, MA, USA), and the cells were harvested by centrifugation (300× *g* for 10 min).

Five amniotic membranes were used to obtain freshly isolated hAMSCs (hAMSC-P0), and 5 additional membranes were used to generate expanded hAMSCs (hAMSC-P2).

Freshly isolated cells (hAMSC/P0) were expanded to passage 2 (hAMSC/P2) in Chang Medium C (Irvine Scientific, Santa Ana, CA, USA) supplemented with 2 mM L-glutamine at 37 °C in 5% CO_2_ incubator.

hAMSCs are plastic adherent cells and were characterized phenotypically both after isolation (P0) and at P2 as previously reported [30] and stored in liquid nitrogen until further use.

Only hAMSC preparations meeting the predefined quality-control criteria were used for this study: viability > 85%, expression of the MSC-associated markers CD13 and CD90 in >80% of cells, and expression of the hematopoietic marker CD45 and epithelial marker CD324 in <10% of cells. These source specific release criteria complement the previously established characterization of hAMSCs performed according to the ISCT framework and consensus recommendations for MSCs derived from fetal membranes [30,31].

### 2.3. Animal Procedures

All experimental procedures were performed on animals anesthetized via intramuscular injection with Tiletamine chloride + zolazepam chloride (Zoletil-100, Virbac, Milano, Italy, at 0.3 mL/30 mg/kg), and xylazine (Ronpum, Bayer Schering Pharma AG, Leverkusen, Germany, at 2 mg/kg). All efforts were made to minimize animal suffering.

Lung injury was induced by intra-tracheal instillation of bleomycin (40 µL; 2.3 U/kg; Bleoprim, Sanofi-Aventis, Milano, Italy) in 8- to 9-week-old female C57BL/6 mice (Charles River, Calco, Italy) [1], control animals were instilled with 40 µL of saline.

After bleomycin challenge, animals were randomly divided into 6 groups (n = 7–9 mice/group): 3 groups received early treatment (15 min after bleomycin instillation) with hAMSC/P0 (Bleo+hAMSC/P0), or hAMSC/P2 (Bleo+hAMSC/P2) or PBS (Bleo+PBS) and 3 groups received the same treatments but at day 7 post-bleomycin (late treatment).

hAMSC/P0 or hAMSC/P2 (4 × 10^6^ cells suspended in 200 µL sterile PBS) were intra-peritoneally injected into each treated mouse. Control mice were instead injected with 200 µL sterile PBS.

In our previous studies, we consistently administered hAMSCs via the intratracheal route. In this specific study, where one treatment arm involved animals already exhibiting pulmonary fibrosis (late treatment), we hypothesized that the potential density of the fibrotic tissue, combined with possible intrapulmonary circulatory occlusion or dysfunction, might result in an uneven distribution of the intratracheally inoculated cells. Conversely, prior experience with intravenous administration (via the caudal vein) in mice had occasionally led to venous occlusion caused by the aggregation of the injected cells. For these reasons, we determined that intraperitoneal administration of hAMSCs was the most suitable and safest approach for the animals’ well-being.

At day 14 post-bleomycin instillation, mice of each experimental group were euthanized by anesthetic overdose and immediately exsanguinated via abdominal aorta.

After bronchoalveolar lavage performed with 2.0 + 0.5 mL of RT PBS, to remove cells present in airway and alveolar spaces, lungs were explanted and sectioned into the five individual lobes which were formalin-fixed and processed for microscopy.

### 2.4. Histological Analyses

Lung lobes were paraffin-embedded and consecutive 4 µm-thick sections were cut. Consecutive sections were stained with hematoxylin and eosin (HE) to evaluate alveolar area or with Masson-Goldner trichrome (Bio-Optica) to evaluate lung fibrosis severity. Sequential, non-overlapping images were captured from HE-stained sections with a digital camera (Mono Camera Nikon DS-Fi3 Version 4.60, Tokyo, Japan) in bright-field light microscopy (Nikon Eclipse Ni-U, Tokyo, Japan) at 200× magnification. Color digital images obtained from each lobe were converted by FiJi software (version 1.54m, https://imagej.nih.gov/ij, accessed on 28 January 2025) to binary data and the percentage of pixels in each alveolar lobe relative to those in the entire lobe was calculated. The area occupied by alveoli of the entire lung was the sum of all lobe alveolar areas and was expressed as a percentage of the total area of the entire lung section [12,32].

Histological grading of fibrosis was performed using a bright-field microscope following a semi-quantitative method and criteria reported by Ashcroft [33]. At least ten randomly chosen, non-overlapping high-power fields (200× magnification) for each section were individually scored for severity of interstitial fibrosis; the mean score of all fields was taken as the fibrosis score for that lobe. For each animal, the fibrosis score of the entire lung was the average of all of the lobes’ mean scores. All analyses were performed in a blinded manner by a veterinary pathologist.

### 2.5. Immunohistochemistry

Serial paraffin-embedded sections of lung tissue (4 μm) were de-waxed and rehydrated in water. Antigen retrieval was performed with 10 mM citrate buffer, pH 8.0 (W-CAP from Bio-Optica, Milano, Italy) or pH6 (Dewax and HIER Buffer from Thermo Fisher Scientific, San Diego, CA, USA) at 98 °C for 5 min. After blocking endogenous peroxidases and phosphatases (Dako Dual Endogenous Enzyme Block; Dako, Glostrup, Denmark) and unspecific binding (10% normal goat serum; Thermo Fisher), sections were single immunostained for α-SMA (1:100; Clone 1A4, Dako) and F4/80 (1:800; GeneTex, Alton Pkwy Irvine, CA, USA), followed by appropriate biotin conjugated secondary antibodies (Vector Laboratories, Inc., Burlingame, CA, USA) and then labeled with Strep-Avidin HRP for α-SMA or with Strep-Avidin AP for F4/80 (Vector Labs).

Immunohistochemical double staining were performed for iNOS (1:500; Clone 4E5, Novus Biologicals, Centennial, CO, USA) and SPARC (1:200; Bioss Antibodies Inc. Woburn, MA, USA), or sialoadhesin (CD169) (1:400; Bioss Antibodies Inc.); and for Arg1 (1:100; Thermo Fisher Scientific) and SPARC (1:200; Bioss Antibodies Inc.), or sialoadhesin (CD169) (1:600; Bioss Antibodies Inc.).

Sections, incubated overnight at 4 °C with one of the primary antibodies, were then washed and after anti-iNOS were incubated with appropriate Alkaline Phosphatase IgG(H+L), (1:500, Vector Laboratories, Inc., Burlingame, CA, USA), and after anti-sialoadhesin (anti-CD169) antibody were incubated or with a horseradish peroxidase (HRP)-conjugated secondary antibody (Vector Laboratories, Inc.) or with Biotinylated, (1:500, Vector Laboratories, Inc.) followed by Horseradish Peroxidase Streptavidin (2 µg/mL, Vector Laboratories, Inc.).

After anti-Arg1 and anti-SPARC antibodies, sections were incubated with appropriate biotin-conjugated secondary antibodies (1:500, Vector Laboratories, Inc.). Anti-Arg1 antibody binding was detected by Alkaline Phosphatase Streptavidin (2 µg/mL, Vector Laboratories, Inc.), and anti-SPARC binding was detected by Horseradish Peroxidase Streptavidin (2 µg/mL, Vector Laboratories, Inc.).

For single and double staining, 5-bromo-4-chloro-3-indolyl-phosphate/Nitro Blue Tetrazolium (BCIP/NBT Alkaline Phosphatase Substrate, Vector Laboratories, Inc, Burlingame, CA, USA) and/or diaminobenzidine (ImmPACT DAB, Vector Laboratories, Inc, Burlingame, CA, USA) were used as chromogens.

Slides were counterstained with hematoxylin and mounted. Substitution of primary antibody with an irrelevant isotype control served as a negative control.

For each single and double staining, at least ten randomly selected images at 200× magnification were taken from each tissue section with a digital camera (Mono Camera Nikon DS-Fi3 Version 4.60). For marker-specific cell counts, cells located in alveolar spaces were excluded from the count.

### 2.6. Immunofluorescence

Other sections of lung tissues, processed as reported in the “Immunohistochemistry” paragraph, were incubated at 4 °C O/N with these specific primary antibodies: anti-iNOS (1:500; Clone 4E5, Novus Biologicals), anti-Arg1 (1:100; Thermo Fisher Scientific), anti-CD169 (1:100; clone B-1; Santa Cruz, Dallas, TX, USA), anti-CX3CR1 (1:50; Bioss, Woburn, MA, USA), anti-IL-10 (1:50; clone JES5-2A5; Abcam, Cambridge, UK) and anti-CCL22 (1:50; Bioss) for macrophage characterization and with anti-CD3 (1:100; A0452; Dako), anti-FOXP3 (1:100; Santa Cruz) and anti-CCR4 (1:50; clone KH-4F5; Santa Cruz) for T lymphocytes and Treg identification.

Immunofluorescent staining was performed with secondary antibodies and control isotype: anti-mouse in horse 594 (DI-2594; 5 µg/mL), anti-rabbit in goat 594 (DI-1594; 5 µg/mL), anti-mouse in horse 488 (DI-2488; 5 µg/mL), anti-rabbit in goat 488 (DI 1488; 5 µg/mL), anti-mouse in horse CY5 (CY-2500; 10 µg/mL) and anti-rat in goat CY5 (CY-4500; 10 µg/mL) all from Vector Laboratories (Burlingame, CA, USA).

Sections were then counterstained and mounted (VECTASHIELD Vibrance Antifade Mounting Medium with DAPI; Vector Laboratories, Inc.).

For marker-specific cell counts, at least ten random high-power fields (200× magnification) were captured using the MICA platform from Leica (Wetzlar, Germany).

### 2.7. Statistical Analysis

All data in the text are reported as mean ± standard deviation (SD). The results in the figures are represented as scatter dot plots with median, where each dot corresponds to an individual mouse. Normality was assessed by Shapiro–Wilk (α = 0.05); normal data were compared by one-way ANOVA followed by Tukey’s multiple comparisons test; non-normal data by Kruskal–Wallis’ test corrected for Dunn’s multiple comparisons test, as reported in each figure.

**Figure 1:**
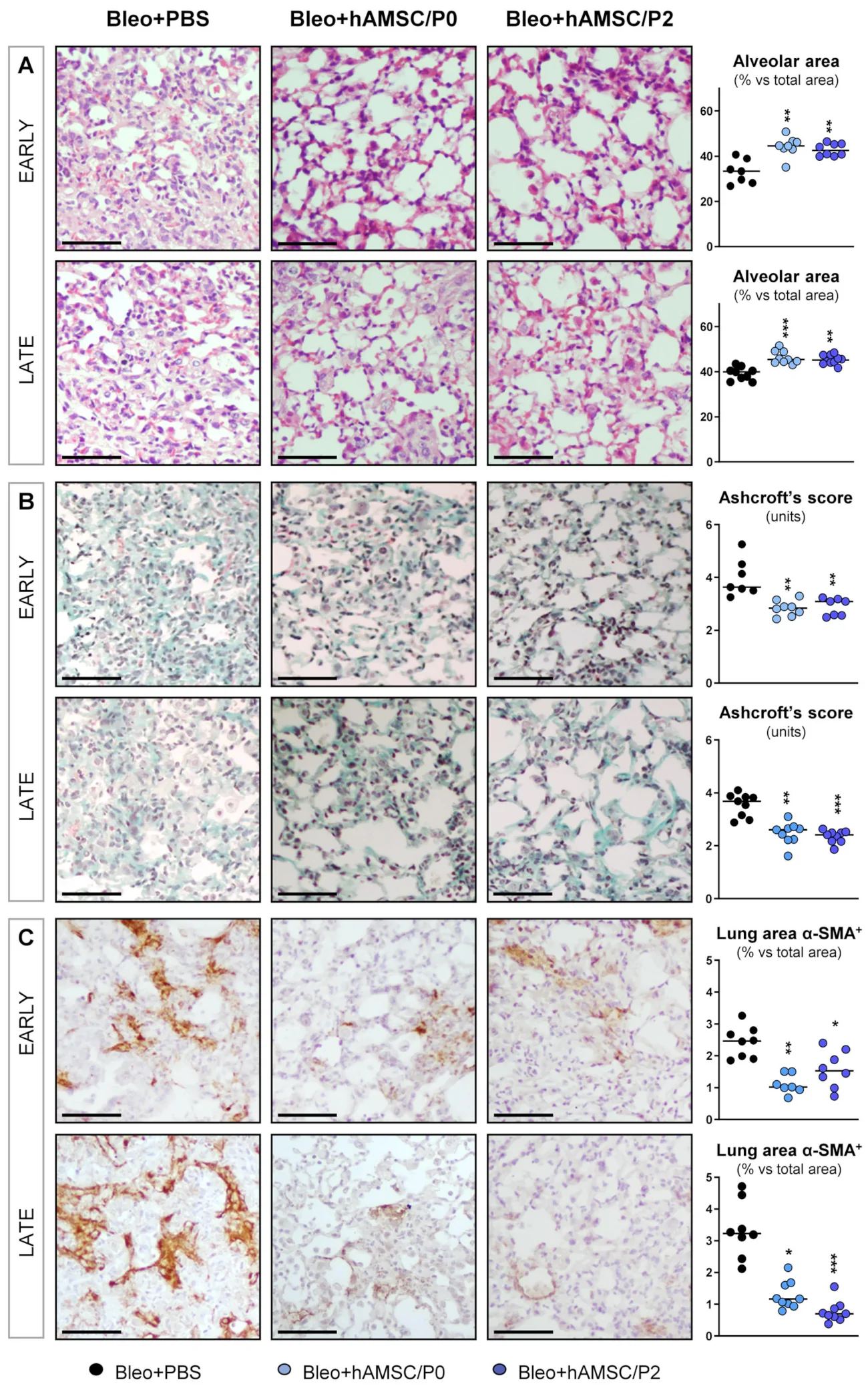
hAMSCs ameliorate bleomycin-induced lung injury after both early and late administrations. Representative photomicrographs of lung sections from control (Bleo+PBS) and treated groups (Bleo+hAMSC P0 and Bleo+hAMSC/P2) are shown, stained with H&E (panels (**A**)), Masson-Goldner trichrome (panels (**B**)), or immunostained for α-SMA (panels (**C**)). For each section, at least ten randomly selected, non-overlapping fields were analyzed. Treatments were given either concomitantly with bleomycin challenge (panels EARLY) or 7 days after bleomycin administration (panels LATE). Lung samples were collected 14 days post-bleomycin instillation to assess residual alveolar area (panels (**A**)-EARLY and (**A**)-LATE), fibrosis severity using Ashcroft’s scoring (panels (**B**)-EARLY and (**B**)-LATE), and α-SMA positive lung area (panels (**C**)-EARLY and (**C**)-LATE). Results represent three independent experiments and are displayed as scatter dot-plots with median, where each dot corresponds to an individual mouse (n = 7–9 per group). Statistical comparisons between groups were made using the Kruskal-Wallis’ test followed by Dunn’s multiple comparisons correction. Statistical significance is indicated as * *p* < 0.05; ** *p* < 0.01; *** *p* < 0.001 compared to control (Bleo+PBS). Scale bars represent 50 μm.

**Figure 2:**
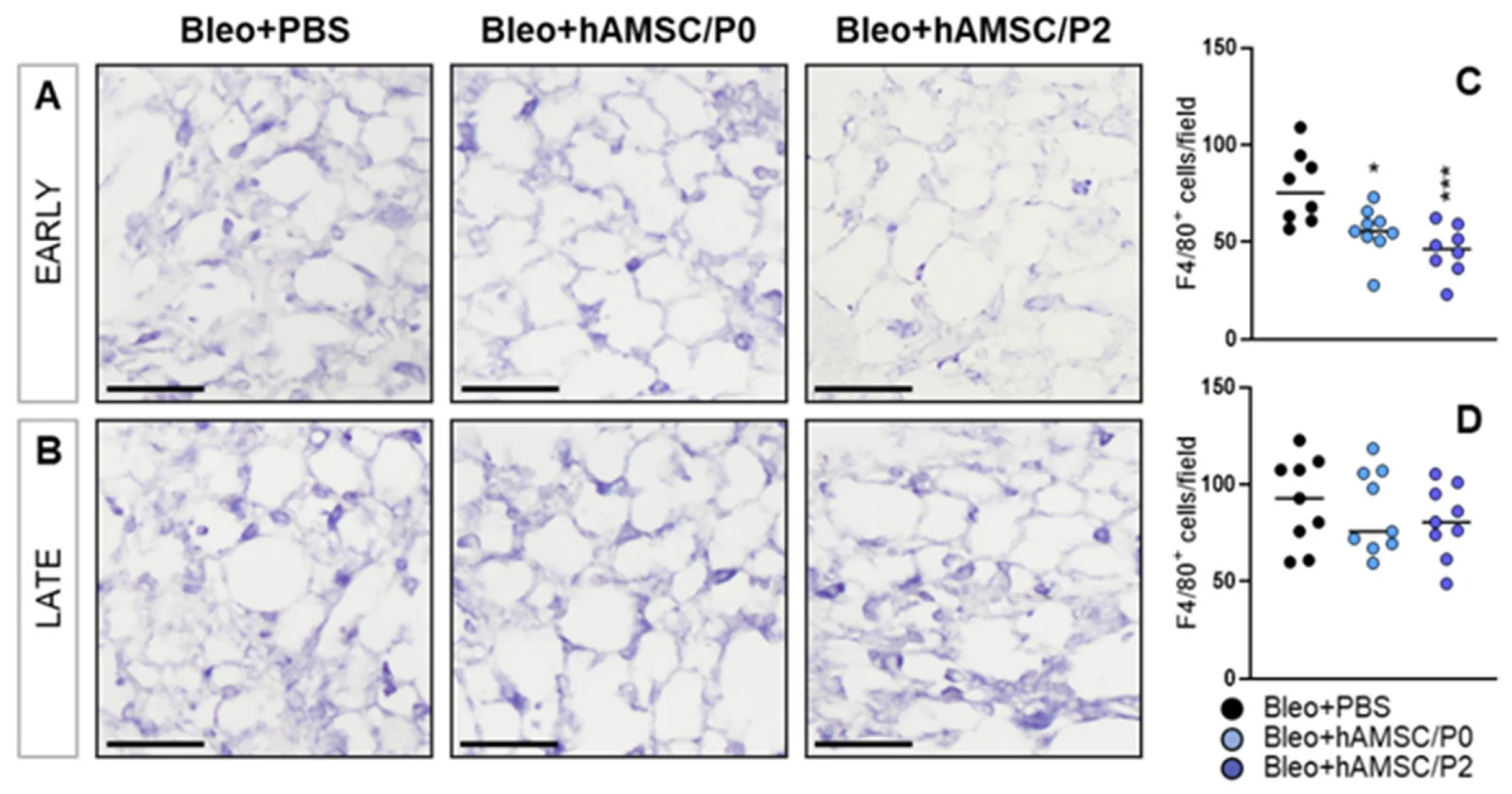
The time of hAMSC administration (early vs. late) affects lung macrophage levels. Representative photomicrographs of lung sections immunostained with anti-F4/80 antibody (pan anti-macrophages) from mice of control (Bleo+PBS) and treated groups (Bleo+hAMSC P0 and Bleo+hAMSC/P2) (panels (**A**,**B**)). Immunodetection was performed using alkaline phosphatase-conjugated secondary antibody and Nitro Blue Tetrazolium as chromogen (see Materials and Methods Section 2.4). For each section, at least ten randomly selected, non-overlapping fields were analyzed. Treatments were administered concomitantly with bleomycin challenge (EARLY), or delivered 7 days post-bleomycin challenge (LATE). The number of F4/80-expressing macrophages/field, after early (panel (**C**)) and late treatment (panel (**D**)), is reported as scatter dot plots with median, where each dot represents 1 mouse (n = 7–9 per experimental group). Between-group comparisons are performed by 1way ANOVA corrected with Tukey’s multiple comparisons test. Statistical significance is indicated as * *p* < 0.05; *** *p* < 0.001 vs. control (Bleo+PBS) group. Scale bars represent 50 μm.

**Figure 3:**
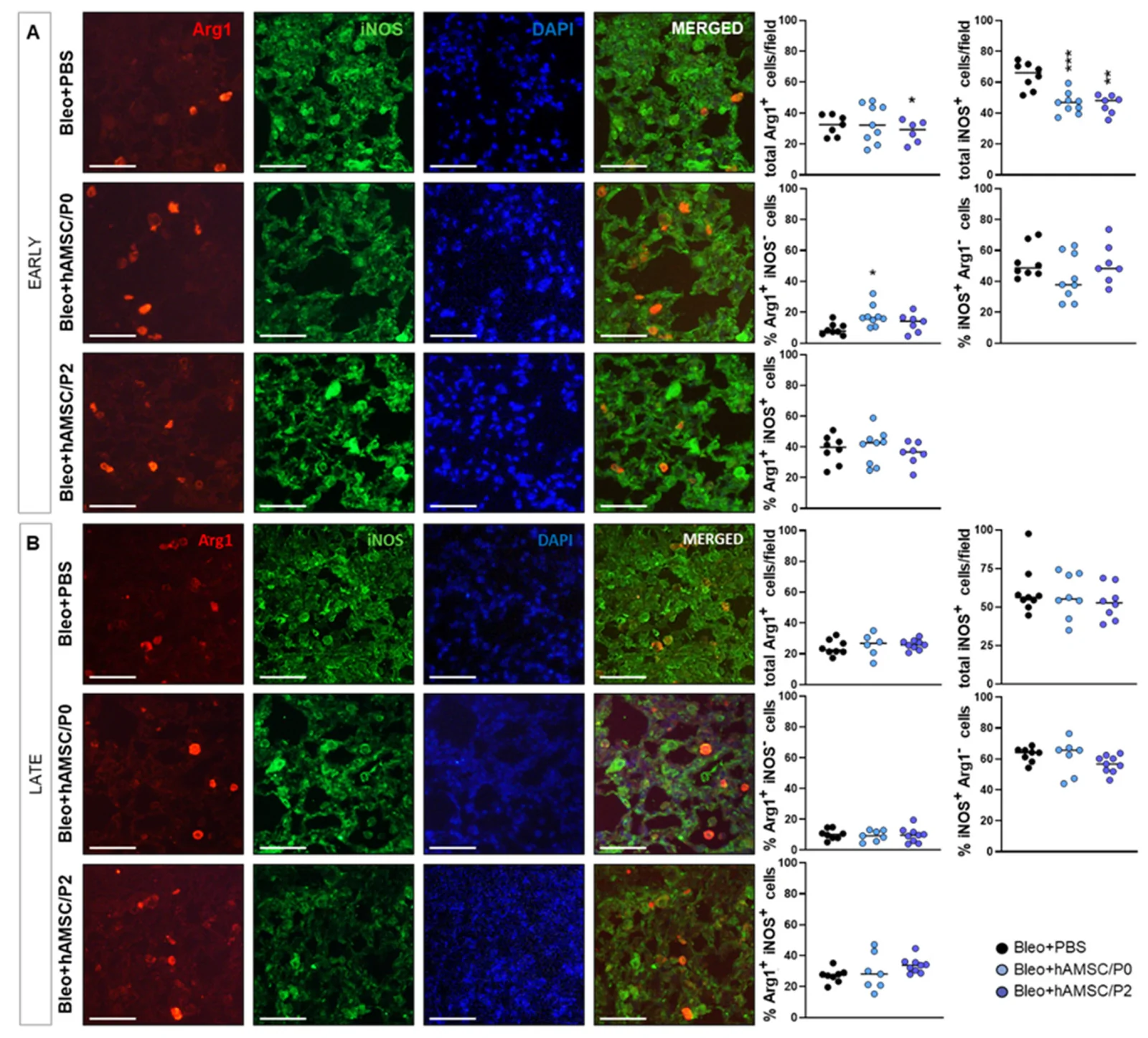
The time of administration influences the effect of the treatment on iNOS- and Arg1-expressing lung macrophages. Representative photomicrographs of lung sections from mice in the control (Bleo+PBS) and treated groups (Bleo+hAMSC P0 and Bleo+hAMSC/P2) at day 14 post-bleomycin instillation. Lung sections were stained using immunofluorescence to visualize the expression of Arg1 (red fluorescence), iNOS (green fluorescence), and the co-expression of Arg1 and iNOS. Nuclear staining was performed using DAPI (4′,6-diamidino-2-phenylindole). For each section, at least ten randomly selected, non-overlapping fields were analyzed. The total number of Arg1- and iNOS-expressing macrophages/field was evaluated after EARLY treatments (administration concomitant with bleomycin challenge) and LATE treatments (administration 7 days post-bleomycin challenge). The relative percentage of double positive cells (Arg1^+^iNOS^+^) and single positive cells (Arg1^+^iNOS^−^ and iNOS^+^Arg1^−^) were calculated with regard to the total numbers of Arg1^+^ and iNOS^+^ cells. Data are reported as scatter dot-plots with median, where each dot represents 1 mouse (n = 7–9 per experimental group). Between-group comparisons were performed by 1way ANOVA corrected with Tukey’s multiple comparisons test. Statistical significance is indicated as * *p* < 0.05; ** *p* < 0.01; *** *p* < 0.001 vs. control (Bleo+PBS) group. Scale bars represent 50 μm.

**Figure 4:**
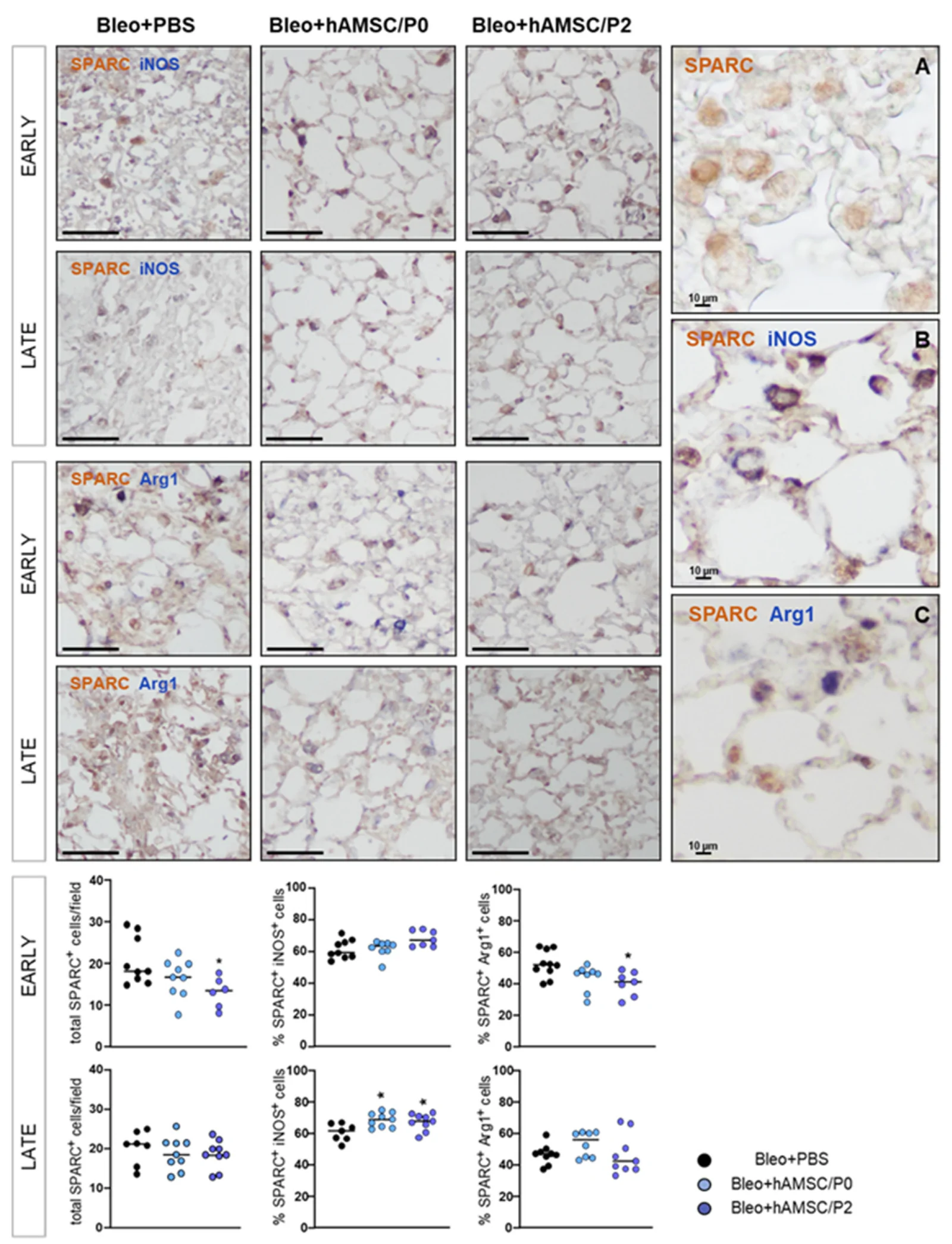
The time of administration influences the effect of the treatment on SPARC-expressing lung macrophages. Representative photomicrographs of lung sections from bleomycin-challenged mice immunostained with anti-SPARC, anti-iNOS and anti-Arg1 antibodies to visualize the macrophage expression of SPARC (panel (**A**)), the co-expression of SPARC/iNOS (panel (**B**)) and of SPARC/Arg1 (panel (**C**)). The total number of SPARC-expressing macrophages/field was evaluated in at least ten randomly selected, non-overlapping fields/section, after EARLY (administration concomitant with bleomycin challenge) and LATE treatments (administration 7 days post-bleomycin challenge). The relative percentage of double positive cells (SPARC^+^iNOS^+^ and SPARC^+^Arg1^+^) was calculated with regard to the total number of SPARC^+^ cells. Data are reported as scatter dot-plots with median, where each dot represents 1 mouse (n = 7–9 per experimental group). Between-group comparisons were performed by 1way ANOVA corrected for Tukey’s multiple comparisons test. Statistical significance is indicated as * *p* < 0.05 vs. control (Bleo+PBS) group. Scale bars represent 50 μm, Except for the magnifications (panels (**A**–**C**)).

**Figure 5:**
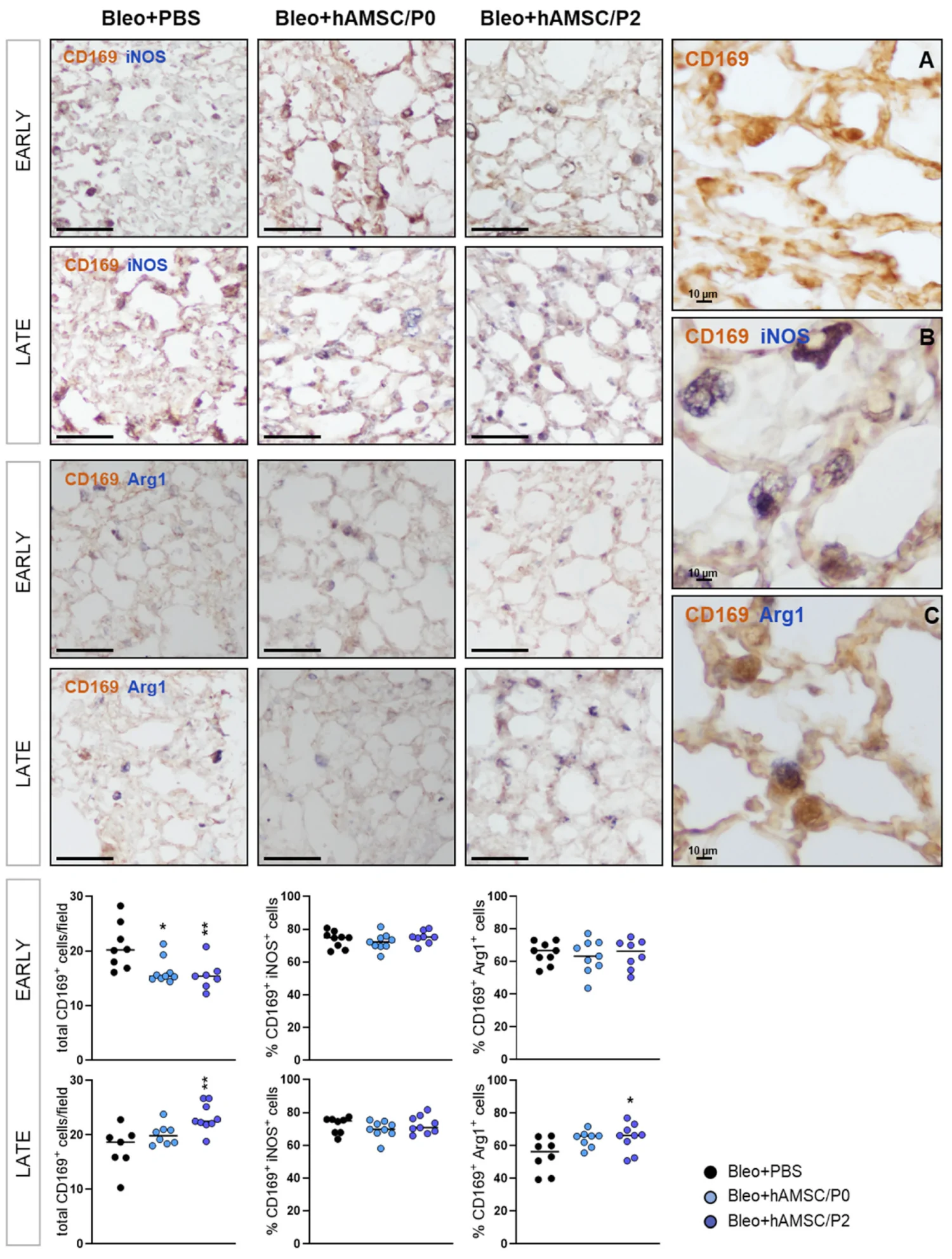
The time of administration influences the effect of the treatment on CD169-expressing lung macrophages. Representative photomicrographs of lung sections from bleomycin-challenged mice immunostained with anti-CD169, anti-iNOS and anti-Arg1 antibodies to visualize the macrophage expression of CD169 (panel (**A**)), the co-expression of CD169/iNOS (panel (**B**)) and of CD169/Arg1 (panel (**C**)). The total number of CD169-expressing macrophages/field was evaluated in at least ten randomly selected, non-overlapping fields/section, after EARLY (administration concomitant with bleomycin challenge) and LATE treatments (administration 7 days post-bleomycin challenge). The relative percentage of double positive cells (CD169^+^iNOS^+^ and CD169^+^Arg1^+^), was calculated with regard to the total number of CD169^+^ cells. Data are reported as scatter dot plots with median, where each dot represents 1 mouse (n = 7–9 per experimental group). Between-group comparisons were performed by 1way ANOVA corrected with Tukey’s multiple comparisons test. Statistical significance is indicated as * *p* < 0.05; ** *p* < 0.01 vs. control (Bleo+PBS) group. Scale bars represent 50 μm. Except for the magnifications (panels (**A**–**C**)).

**Figure 6:**
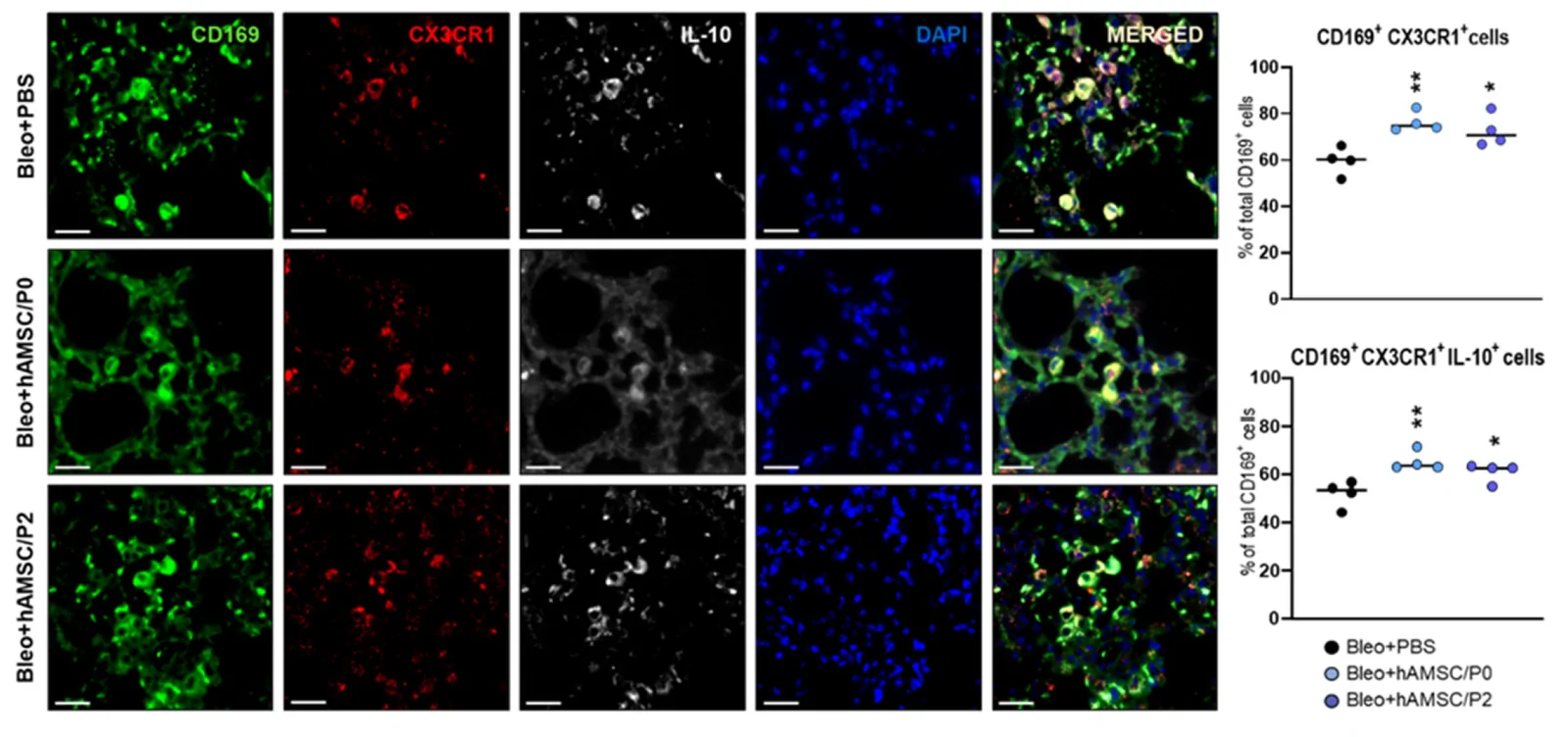
CD169-expressing lung macrophages also express CX3CR1 and IL-10. Representative photomicrographs of lung sections from bleomycin-challenged mice stained using immunofluorescence to visualize CD169 (green), CX3CR1 (red) and IL-10 (pseudo-colored in white) cell expression and co-expression. The relative percentage of cells double positive for CD169 and CX3CR1 and triple positive for CD169, CX3CR1 and IL-10 are evaluated after late treatments (administration 7 days post-bleomycin challenge) in at least ten randomly selected, non-overlapping fields/section. Data are reported as scatter dot plots with the median, where each dot represents 1 mouse (n = 4 per experimental group). Between-group comparisons were performed by 1way ANOVA corrected with Tukey’s multiple comparisons test. Statistical significance is indicated as * *p* < 0.05; ** *p* < 0.01 vs. control (Bleo+PBS) group. Scale bars represent 50 μm.

**Figure 7:**
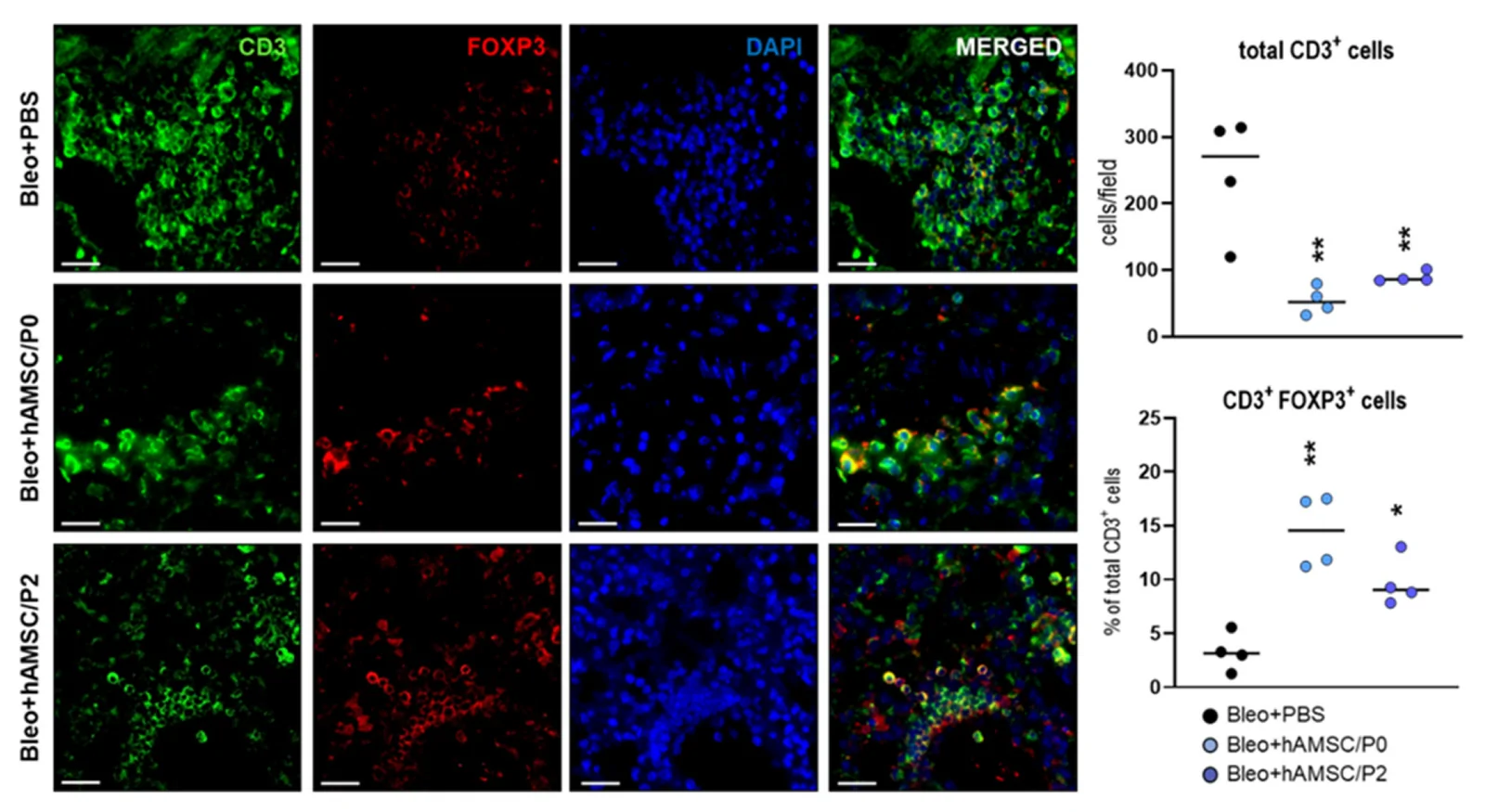
Effect of late treatment on lung Treg (CD3^+^ cells expressing FOXP3). Representative photomicrographs of lung sections from bleomycin-challenged mice stained using immunofluorescence to visualize CD3 (green) and FOXP3 (red) cell expression and co-expression. The total number of CD3-expressing cells/field was evaluated in at least ten randomly selected, non-overlapping fields/section after late treatment (administration 7 days post-bleomycin challenge). The relative percentage of double-positive cells for CD3 and FOXP3 was calculated with regard to the total number of CD3^+^ cells. Data are reported as scatter dot-plots with median, where each dot represents 1 mouse (n = 4 per experimental group). Between-group comparisons were performed by 1way ANOVA corrected with Tukey’s multiple comparisons test. Statistical significance is indicated as * *p* < 0.05; ** *p* < 0.01 vs. control (Bleo+PBS) group. Scale bars represent 50 μm.

**Figure 8:**
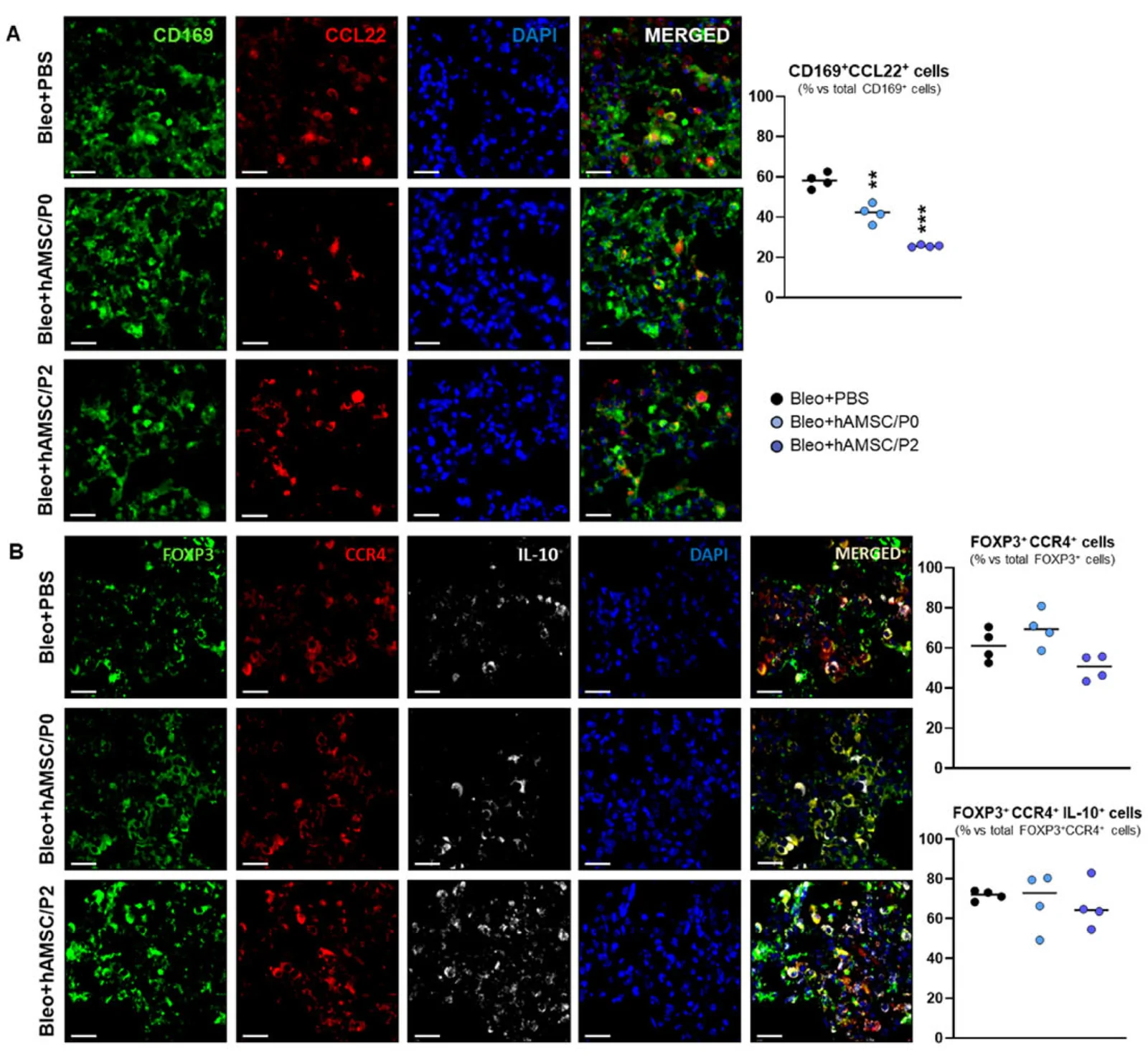
Effect of late treatment on lung macrophage/Treg (CCL22/CCR4) axis. Panel (**A**) shows representative photomicrographs of lung sections from bleomycin-challenged mice stained using immunofluorescence to visualize CD169 (green) and CCL22 (red) cell expression and co-expression. The relative percentage of cells double positive for CD169 and CCL22 is evaluated after late treatments (administration 7 days post-bleomycin challenge) in at least ten randomly selected, non-overlapping fields/section. Panel (**B**) shows representative photomicrographs of lung sections from bleomycin-challenged mice stained using immunofluorescence to visualize FOXP3 (green), CCR4 (red) and IL-10 (pseudo-colored in white) cell expression and co-expression. The relative percentage of cells double positive for FOXP3 and CCR4 and triple positive for FOXP3, CCR4 and IL-10 are evaluated after late treatments (administration 7 days post-bleomycin challenge) in at least ten randomly selected, non-overlapping high-power fields/section. Data are reported as scatter dot plots with median, where each dot represents 1 mouse (n = 4 per experimental group). Between-group comparisons were performed by 1way ANOVA corrected with Tukey’s multiple comparisons test. Statistical significance is indicated as ** *p* < 0.01; *** *p* < 0.001 vs. control (Bleo+PBS) group. Scale bars represent 50 μm.

For the primary outcomes (Figure 1, Figure 2, Figure 3, Figure 4 and Figure 5), sample sizes (n = 7–9) were based on historical laboratory data. Conversely, the mechanistic evaluation of CD169^+^ macrophages and Treg recruitment (Figure 6, Figure 7 and Figure 8) was designed as a focused, hypothesis-generating subset; a sample size of (n = 4) per group was adopted. No a priori power calculations were performed for this exploratory phase, and data should be interpreted as observational.

A *p*-value < 0.05 was considered statistically significant. Statistical analyses were performed using Prism 9.5.1 software (GraphPad software Inc., La Jolla, CA, USA).

## 3. Results

### 3.1. hAMSC Effect on Bleomycin-Induced Lung Fibrosis

We evaluated the effect of hAMSC treatment on bleomycin-induced lung injury by assessing the residual alveolar area, the severity of lung fibrotic lesions (through Ashcroft’s score system), and the lung accumulation of α-SMA^+^ myofibroblasts, which are mainly responsible for collagen deposition (Figure 1).

At 14 days post-instillation, control fibrotic mice (Bleo+PBS group) displayed significant lung inflammation accompanied by extensive collagen deposition, resulting in thickening of the interstitial septa, partial alveolar obliteration, and distortion of the normal lung architecture. Compared to healthy control mice, the fibrotic mice exhibited: (a) reduced lung alveolar area (from 58.6 ± 3.5, in healthy control PBS-instilled mice, to 33.1 ± 2.0 and to 39.2 ± 1.0%, in Bleo+PBS early and late groups, respectively; *p* < 0.01); (b) increased Ashcroft’s score (from 0.91 ± 0.56, in healthy control PBS-instilled mice, to 3.98 ± 0.70 and to 3.54 ± 0.15 units, in Bleo+PBS early and late groups, respectively; *p* < 0.01) and (c) enlarged lung area positive for α-SMA (from 0.61 ± 0.42, in healthy control PBS-instilled mice, to 2.43 ± 0.17 and to 3.33 ± 0.31 in Bleo+PBS early and late groups, respectively; *p* < 0.01).

hAMSCs administered at the time of injury induction (early treatment) limited the reduction in alveolar area caused by bleomycin exposure, mitigated the extent of lung fibrosis, and simultaneously lowered the lung area positive for α-SMA expression (Figure 1A–C-EARLY).

Notably, hAMSC treatment is effective in halting the progression of lung fibrosis even when administered 7 days after bleomycin challenge (late treatment), when lung fibrosis is already established, as confirmed by histological analyses of lungs from an additional group of Bleo+PBS-treated mice sacrificed at that time.

At 7 days post-bleomycin, these mice showed a decline in residual alveolar area from 58.6 ± 3.5% to 41.1 ± 3.1% (*p* < 0.01) and an increase in fibrosis score from 0.91 ± 0.56 to 2.94 ± 0.90 Ashcroft’s units (*p* < 0.01) compared to healthy control PBS-instilled controls, confirming the presence of lung fibrosis.

hAMSC treatment, even when administered late, preserved the residual alveolar area and stopped the fibrosis score deterioration and the expansion of lung area positive for α-SMA expression (Figure 1A–C-LATE).

Overall, these data show that treatment with either freshly isolated (hAMSC/P0) and expanded (hAMSC/P2), prevents and halts lung injury in bleomycin-challenged mice, depending on the time of administration. Notably, the amelioration of lung conditions is associated with an improvement in the general health of the animals. By the end of the experiment, mice treated with hAMSCs—regardless of cell passage (P0 or P2) or timing of administration (early and late)—showed significantly increased body weight. In contrast, mice of the relative control groups (Bleo+PBS) exhibited a net weight loss (Figure S1).

### 3.2. hAMSC Effect on Lung Macrophage Levels

To assess whether amniotic cells’ anti-fibrotic effects are associated with changes in lung macrophage populations, we first examined bleomycin and treatment impact on macrophage recruitment. Using F4/80 as a pan-macrophage marker [34], we observed pronounced macrophage infiltration in the lung parenchyma of mice after intratracheal bleomycin instillation. Specifically, F4/80-positive cells rose significantly from 4.89 ± 1.17 cells/field in healthy control PBS-instilled mice to 85.5 ± 21.4 cells/field in the bleomycin-treated (Bleo+PBS) group.

We next tested whether hAMSC treatment could modulate macrophage levels. Early treatment (at bleomycin challenge) with both hAMSC/P0 and hAMSC/P2 significantly limited macrophage accumulation (Figure 2A,C), whereas late treatment (7 days post-bleomycin) had no effect on macrophage accumulation (Figure 2B,D).

### 3.3. hAMSC Effect on Lung Macrophages Expressing iNOS and Arg1

Next, we investigated whether treatment and treatment timing differentially affected macrophage polarization. Macrophages can adapt their functions in response to different tissue microenvironments. By modulating the expression of inducible nitric oxide synthase (iNOS) and arginase-1 (Arg1), macrophages shift their arginine metabolism toward either a pro-inflammatory, defensive role (mediated by nitric oxide production) or a reparative/anti-inflammatory role (via ornithine production) [35,36].

Although the expressions of iNOS and Arg1 are commonly used as markers to identify distinct macrophage M1 and M2 populations with distinct pro-inflammatory and anti-inflammatory roles, these markers are not exclusively expressed by these single subpopulations. Rather, macrophages can co-express both, reflecting various stages of maturation and activation [15].

Accordingly, beyond assessing lung levels of macrophages with individual Arg1- and iNOS-expression, we also examined their co-expression. Consistent with prior findings, bleomycin-treated mice lungs contained cells expressing these markers separately or simultaneously (Figure S2).

Specifically, when evaluating the expression of iNOS and Arg1 regardless of whether they were expressed individually or in combination, we observed that early treatment decreased the number of iNOS-positive and Arg1-positive lung macrophages (Figure 3A). Within the total cell macrophage population, the relative proportions of cells expressing iNOS alone (iNOS^+^ Arg1^−^) or co-expressing iNOS with Arg1 (iNOS^+^ Arg1^+^) remained unchanged. However, the proportion of macrophages expressing Arg1 alone (Arg1^+^ iNOS^−^) tended to increase, suggesting that the early treatment promotes a shift in lung macrophages from pro-inflammatory nitric oxide production to Arg1-mediated anti-inflammatory ornithine production.

In contrast, late hAMSC treatment did not change the total number of lung macrophages expressing iNOS and Arg1, regardless of whether these markers were expressed individually or in combination, nor did it alter the relative proportions of macrophages expressing iNOS alone (iNOS^+^ Arg1^−^), Arg alone (Arg1^+^ iNOS^−^) or both markers (iNOS^+^ Arg1^+^) (Figure 3B).

### 3.4. hAMSC Effect on Lung Macrophages Expressing SPARC

Considering that SPARC-expressing macrophages can play contrasting roles in fibrosis, either by contributing to ECM deposition [37] or, conversely, reducing lung inflammation and fibrosis [21] and participating in ECM degradation [25], we evaluated SPARC expression in lung macrophages of bleomycin-challenged mice and explored the effect of treatment on their lung levels.

We found that bleomycin instillation significantly increased the number of SPARC-positive macrophages in the lungs, rising from 1.67 ± 0.33 cells/field in healthy control PBS-instilled mice to 19.2 ± 3.9 cells/field in the bleomycin-challenged (Bleo+PBS) group.

Furthermore, we observed that treatment timing impacts SPARC-expressing macrophages. Early treatment, mainly with hAMSC/P2, reduced the total number of these lung macrophages (Figure 4, EARLY panel), while late treatment did not affect their overall number (Figure 4, LATE panel).

As early treatment restrains bleomycin-driven accumulation of SPARC^+^ macrophages while concurrently exerting anti-fibrotic effects, this suggests these cells predominantly drive pro-fibrotic rather than anti-fibrotic mechanisms.

Moreover, since SPARC uptake and degradation are limited to alternatively activated (M2) macrophages [25] and SPARC can influence macrophage polarization, favoring a shift from anti-inflammatory to pro-inflammatory states [38], we also explored whether SPARC expression is associated with Arg1 and/or with iNOS expression.

In this context, our data indicate that SPARC may be co-expressed with both Arg1 and iNOS (Figure 4A–C) and that the early treatment decreased the percentage of SPARC^+^Arg1^+^ co-expressing cells (Figure 4, EARLY panel), while late treatment increased the percentage of SPARC^+^iNOS^+^ co-expressing cells (Figure 4, LATE panel).

These data suggest that, despite lowering SPARC^+^ macrophage levels in the lung, treatment cannot counteract SPARC-driven polarization toward a pro-inflammatory phenotype.

### 3.5. hAMSC Effect on Lung Macrophages Expressing Sialoadhesin (CD169)

Macrophages expressing CD169 have also been recently detected in lung parenchyma around the large bronchiolar airways and adjacent to airway-associated nerves [22]. Considering the potential role of lung interstitial macrophages expressing CD169 in slowing down lung inflammation and restraining the fibrotic process [22], we evaluated whether hAMSC treatment affects their levels in the lungs of bleomycin-challenged mice and whether this effect depends on treatment timing.

We found that bleomycin instillation increased the lung levels of CD169-expressing macrophages. The number of CD169-positive cells in the lung parenchyma of PBS-instilled healthy control mice was 6.00 ± 1.45 cells/field and 19.05 ± 4.4 cells/field in bleomycin-challenged mice (Bleo+PBS group). Interestingly, the levels of CD169-expressing macrophages are affected in opposite ways by hAMSC treatment, depending on the timing of administration.

Early administration of hAMSCs reduced the total number of CD169^+^ macrophages in the lungs (Figure 5, panel EARLY), while late administration increased their levels (Figure 5, panel LATE).

In addition, since little is known about the function of CD169-expressing macrophages, especially in the lung, we evaluated whether these cells exhibit a specific pro- or anti-inflammatory phenotype by examining CD169 co-expression with Arg1 and iNOS.

We observed that CD169 macrophages may co-express both Arg1 and iNOS markers (Figure 5A–C) and that early treatment did not modify the proportions of CD169^+^ macrophages co-expressing Arg1 (CD169^+^Arg1^+^) and iNOS (CD169^+^iNOS^+^) (Figure 5, panel EARLY), while late treatment increased the percentage of CD169^+^ macrophages that co-express Arg1 (CD169^+^Arg1^+^) (Figure 5, panel LATE).

The decrease in CD169^+^ macrophages following early treatment seems consistent with the reduced macrophage recruitment observed using the pan-macrophage marker F4/80 (see Figure 2). In contrast, the increase in the number of these cells and their shift toward a more anti-inflammatory phenotype after late treatment suggests their possible involvement in the anti-inflammatory and anti-fibrotic effects exerted by hAMSC therapy.

### 3.6. Lung CD169^+^ Macrophage Phenotype

Previous studies have evidenced the crucial role of an interstitial subset of CD169^+^ lung-resident macrophages in regulating lung inflammation in vivo [22]. This macrophage subset, named NAMs, shares CX3CR1 expression with interstitial macrophages and exhibits a regulatory profile via IL10 expression and secretion.

We investigated whether the CD169-expressing cells found in the lungs of bleomycin-challenged mice share similar markers with NAMs, in particular the co-expression of CX3CR1 and IL-10. Moreover, we examined whether late treatment, which was previously shown to increase the levels of CD169-expressing macrophages in the lung, also affects the CD169-positive cells co-expressing CX3CR1 and IL-10.

We observed that a large proportion of CD169-positive cells in the lungs of control bleomycin-challenged mice (Bleo+PBS group) also express CX3CR1 (59.7 ± 5.9% of total CD169^+^ cells) and both CX3CR1 and IL-10 simultaneously (51.8 ± 5.4% of total CD169^+^ cells) (Figure 6). Notably, late treatment significantly increased the percentage of CD169^+^ macrophages co-expressing CX3CR1 to 76.8 ± 4.3 and 72.6 ± 6.9% in groups treated with hAMSC/P0 and hAMSC/P2, respectively (Figure 6). Similarly, late treatment increased the proportion of CD169^+^ macrophages co-expressing both CX3CR1 and IL-10 to 65.3% ± 4.0 and 60.9% ± 4.0 in the hAMSC/P0 and hAMSC/P2 groups, respectively (Figure 6).

Our results indicate that late treatment leads to higher levels of CD169^+^ macrophages that exhibit some phenotypic traits associated with NAMs in the lungs of bleomycin-challenged mice.

### 3.7. hAMSC Effect on T Regulatory Cells

We have shown that, in bleomycin-challenged mice, hAMSC late treatment is able to increase lung levels of CD169^+^ macrophages, which exhibit some phenotypic traits associated with NAMs. However, the functional role of CD169^+^ interstitial macrophages in lung homeostasis and disease remains unclear [20,22]. Previous studies have shown that these cells drive apoptotic cell clearance, inducing the macrophagic expression of the chemokine CCL22. This, acting as a chemoattractant for CCR4-expressing regulatory T cells (Tregs), establishes a CCL22-CCR4 signaling axis [29], which has been shown to contribute to immune tolerance [29,39,40,41] and protection against inflammatory-driven tissue injury [42,43].

In this scenario, late treatment with hAMSCs, by increasing the levels of CD169^+^ macrophages in the lungs, could promote long-term control of inflammation through the recruitment of Tregs, thereby preventing the progression of inflammation-driven fibrosis.

First, we verified whether late treatment with hAMSCs increased lung Treg levels.

Our data show that late treatment strongly reduced lung infiltration of T lymphocytes (total CD3^+^ cells), from 243.9 ± 90.5 in the Bleo+PBS group to 53.8 ± 20.6 and 89.1 ± 8.1 cells/field in groups treated with hAMSC/P0 and hAMSC/P2, respectively (Figure 7). Interestingly, FOXP3^+^ T cells in the lungs of treated mice did not decrease but instead increased (from 6.4 ± 1.7 in the Bleo+PBS group to 8.4 ± 2.5 and 8.6 ± 1.7 cells/field in groups treated with hAMSC/P0 and hAMSC/P2, respectively), resulting in a significantly higher proportion of FOXP3^+^ cells among total CD3^+^ T cells (Figure 7). Thus, late treatment effectively increased lung Treg levels.

### 3.8. Contribution of CD169^+^ Macrophages to the Establishment of a CCL22/CCR4 Axis with Treg Cells

To investigate the possible contribution of the CCL22/CCR4 axis to the effect of hAMSC on Treg levels, we then verified whether late treatment could increase the lung proportion of CD169^+^ macrophages expressing CCL22 and of Treg cells expressing CCR4.

Our data show that more than half of the CD169-expressing cells also express CCL22 (58.2 ± 3.8% in mice of Bleo+PBS group, Figure 8A); similarly, more than half of the FOXP3-expressing cells co-express CCR4 (61.4 ± 4.1% in mice of Bleo+PBS group, Figure 8B). Among these cells, double-positive for FOXP3 and CCR4, a high percentage also express IL10 (71.6 ± 2.5% in mice of Bleo+PBS group, Figure 8B), suggesting an IL10-mediated immunomodulatory action by these cells. These data suggest that CD169^+^ macrophages can establish a CCL22-CCR4 signaling axis potentially responsible for Treg recruitment in lungs. However, although late treatment with hAMSCs increased Treg levels in lungs of bleomycin-challenged mice, this effect does not appear to act through enhanced levels of CD169^+^ macrophages. Indeed, late treatment did not increase but significantly reduced the proportion of CD169^+^ macrophages co-expressing CCL22 (Figure 8A), and it did not change the proportion of Tregs expressing CCR4 and IL10 (Figure 8B).

## 4. Discussion

Previous studies from our group have demonstrated the ability of hAMSCs to prevent bleomycin-induced lung fibrosis [1,2]. In the present study, we confirm that both freshly isolated (hAMSC/P0) and in vitro expanded (hAMSC/P2) hAMSCs exert significant anti-fibrotic effects when administered concomitantly with injury induction (early treatment) or in mice with established fibrosis at day 7 post-bleomycin (late treatment). These findings extend prior observations by showing that hAMSC therapy not only prevents fibrosis progression but also can arrest a progressive pre-existing fibrotic condition.

Although we have transplanted xenogeneic cells, our prior studies revealed that hAMSCs systemically (IP) delivered in immune-competent mice reach lung tissue and persist for 2 weeks, but with low engraftment insufficient to explain anti-fibrotic effects via tissue replacement [1]. This suggested that hAMSCs, like MSCs generally, act via paracrine signaling, confirmed by equivalent preventive anti-fibrotic activity exerted by the hAMSC secretome [44,45]. However, in this study, to investigate a potential therapy for established fibrosis, we used cells, not the secretome, as our preliminary data showed the secretome failed to limit established fibrosis, unlike cells. This suggests that the inflammatory environment, induced by bleomycin, acts as a priming signal for the injected hAMSCs, which dynamically modulate their secretome in response to the local fibrotic niche. Consequently, it is reasonable to assume that the secretome from cells not exposed to this specific milieu cannot produce an equivalently targeted therapeutic effect.

The ability of hAMSCs to halt the progression of established lung fibrosis confirms previous data from the same bleomycin-induced pulmonary fibrosis model. In these studies, cells were administered 7, 10 or 14 days post bleomycin instillation, using amniotic epithelial cells [46] or, more recently, perinatal-derived MSCs (including those from Wharton’s jelly and chorionic villi) [47].

Notably, our data, collected by applying treatment at 2 different stages of disease (inflammatory and fibrotic), provide an original contribution by demonstrating that the preventive and late anti-fibrotic effects of hAMSCs are associated with context-dependent changes in macrophage signatures.

Early treatment, with either hAMSC/P0 or hAMSC/P2, significantly reduced total macrophage accumulation in the lungs, including iNOS^+^, SPARC^+^ and CD169^+^ macrophages. This suggests that treatment primarily acts by limiting macrophage/monocyte recruitment at the interstitial level.

In parallel, early treatment lowered both the total number of iNOS- and Arg1-expressing macrophages. Studies in murine models have usually considered iNOS as a marker of pro-inflammatory M1-like macrophages and Arg1 as a marker of anti-inflammatory/wound repair M2-like macrophages [48,49]. Since several studies have shown that macrophage differentiation into M2-like macrophages correlates with fibrosis progression and severity in bleomycin-induced pulmonary fibrosis [50,51], the observed reduction in Arg1^+^ macrophages could be in line with the treatment’s anti-fibrotic effect.

However, other studies reported increased M2-like macrophages after anti-fibrotic treatments, such as a lipoxin A4 analog [52] or perinatal cells, including amniotic- and umbilical cord-derived cells, in both pulmonary fibrosis [10,12,53] and liver fibrosis [54].

These discrepancies highlight the dual pro-fibrotic and anti-fibrotic roles of M2-like macrophages, depending on the tissue microenvironment. They promote tissue regeneration by resolving inflammation and initiating fibrotic repair, which they keep under control by secreting matrix metalloproteinases to degrade extracellular matrix during fibrosis resolution. Yet, in persistent lesions, M2 macrophages can activate resident fibroblasts and drive fibrosis [55].

These inconsistencies may also stem from the oversimplified M1/M2 classification based on markers like iNOS for M1 and Arg1 for M2 without accounting for the fact that, as our data indicate, lung interstitial macrophages, like those in other tissues [56,57], exhibit a plastic phenotype with mixed expression of the two markers (e.g., double-positive for iNOS and Arg1).

Thus, reanalysis of iNOS and Arg1 co-expression provided key insights: early treatment strongly reduced iNOS^+^ macrophages (with or without Arg1). Conversely, while early treatment decreased total Arg1^+^ macrophages (with or without iNOS), it increased those expressing only Arg1 (without iNOS). Since this Arg1^+^ iNOS^−^ was the only macrophage population that expanded after early treatment, when all others decreased, these findings suggest that hAMSC-induced polarization toward these cells promotes an anti-inflammatory, wound-healing effect [10,58]; rather than the pro-fibrotic role reported in other studies [59,60].

It is also noteworthy that early hAMSC treatment reduces the number of macrophages producing and secreting SPARC in lung tissue. This effect may contribute to lower early macrophage recruitment, as SPARC^+^ macrophages help regulate the assembly and maintenance of ECM fibers, essential for initial immune cell access and migration to injury or infection sites [61,62].

Unlike early treatment, late delivery of hAMSCs neither reduced macrophage lung recruitment nor altered the proportions of SPARC^+^ or Arg1^+^/iNOS^+^ macrophages. This highlights a fundamental difference in the therapeutic mechanism once the disease enters the fibrotic phase. For the first time, we observed that the late anti-fibrotic effect of hAMSCs is associated with a significant increase in interstitial CD169-expressing macrophages and specifically of the double-positive CD169^+^Arg1^+^ macrophages. These macrophages co-express CX3CR1 and IL-10, some phenotypic features of NAMs [22]. NAMs exhibit a regulatory genetic profile and play a crucial role in controlling lung inflammation in vivo, likely via secretion of the immunosuppressive factor IL-10 [22]. Recent findings further show that lung CX3CR1^+^ interstitial macrophages prevent bacterial dysbiosis-driven inflammation and pulmonary fibrosis via the IL-10/IL-10 receptor axis [63]. Supporting a potential anti-fibrotic role of CD169^+^ macrophages, macrophages with a partially overlapping phenotype to NAMs exert anti-fibrotic action in a model of bleomycin-induced pulmonary fibrosis [20].

The functional role of CD169^+^ interstitial macrophages in lung homeostasis and disease remains incompletely defined. Based on evidence that bleomycin induces apoptosis in pulmonary cells [64] and that splenic CD169^+^ macrophages, involved in apoptotic cell clearance, can promote Treg migration and activation via CCL22 [29], we hypothesized that lung CD169^+^ macrophages may contribute to apoptotic cell clearance while promoting lung Treg cell recruitment through the CCL22/CCR4 axis. In this scenario, late treatment with hAMSCs, by increasing the levels of CD169^+^ macrophages in the lungs, could promote long-term control of inflammation through the recruitment of Tregs, thereby preventing the progression of inflammation-driven fibrosis. In this context, we found that although late treatment reduced total CD3^+^ T lymphocyte lung infiltration, it increased FOXP3^+^ Treg levels in the lungs. Additionally, over half of CD169^+^ macrophages expressed CCL22, and over half of FOXP3^+^ cells expressed CCR4, supporting a potential establishment of a CCL22-CCR4 signaling axis for Treg recruitment. However, late treatment reduced the proportion of CCL22-expressing CD169^+^ macrophages without changing the proportions of CCR4^+^ or IL-10^+^ Tregs, suggesting that the observed Treg increase is not directly explained by CD169-driven CCL22 at the specific time point analyzed. One possibility is that the sampling time (day 14 post-bleomycin) captured a descending phase of recruitment and missed an earlier peak of CCL22 upregulation that preceded Treg accumulation.

It is important to note that the increase in Tregs observed after late treatment may involve cellular interactions beyond CD169^+^ macrophages in the tissue microenvironment. Recent evidence shows that CCL22 expression promotes direct physical contact between dendritic cells (DCs) and Tregs, which is essential for Treg activation and immunosuppressive function [65].

Conversely, CCL17 production by DCs restricts Treg expansion, compromising their homeostatic maintenance while favoring a T cell effector response [66].

These insights, combined with our data, indicate that strategies to enhance the CCL22-CCR4 axis and inhibit CCL17 activity could restore Treg balance and reduce inflammation. We acknowledge that the findings regarding CD169^+^ macrophages and Treg lung homing were obtained from a smaller cohort of animals. While this low sample size limits definitive confirmatory conclusions, this exploratory analysis offers valuable preliminary insights that lay the groundwork for future dedicated research.

Altogether, our data suggest that the anti-fibrotic action of hAMSCs is associated with an anti-inflammatory and reparative milieu which may involve acting on different macrophage populations depending on the stage of the disease at which hAMSC administration occurred.

The administration of hAMSCs concomitantly with the onset of the inflammatory phase generally reduces macrophage recruitment and promotes their polarization toward an anti-inflammatory phenotype, preventing the release of inflammatory factors and further activation of immune cells and fibroblasts, ultimately curbing myofibroblast formation and EMT accumulation.

The administration of hAMSCs during the fibrotic phase, instead, does not impact the injury-induced acute inflammatory reaction, but we found for the first time an increase in polarization toward CD169-positive macrophages that could be involved in promoting Treg lung recruitment.

The dual-timing design of this study allowed the observation that early vs. late treatment engages possibly different immunomodulatory anti-fibrotic pathways.

Our results may inform the design of future translational studies investigating whether disease stage influences immunomodulatory effects of hAMSC therapy.

However, this is an observational study; therefore, we underline the correlative nature of our results and that functional validation experiments (for example, adoptive transfer of ex vivo polarized CD169^+^ macrophages into bleomycin-challenged mice) should be necessary to establish functional causality. In this study, macrophage signatures were determined by marker expression and co-expression using immunohistochemistry and immunofluorescence (IHC/IF). A major advantage of IHC/IF is that it preserves spatial relationships within intact tissue architecture and avoids the need for tissue dissociation protocols, thereby minimizing artefacts that can distort the apparent proportions and phenotypes of macrophage subsets. Nevertheless, high-parameter approaches (for example, spectral flow cytometry, CyTOF, single-cell RNA sequencing, and spatial transcriptomics) will be useful to confirm macrophage subpopulation identities, to resolve additional subsets (e.g., CD206^+^, CD86^+^, MHC-II^+^, Ly6C^+^, CCR2^+^, CD11b^+^, CD11c^+^), and will strengthen the mechanistic interpretation and translational relevance of this study.

Additionally, our findings show that expanded hAMSCs (hAMSC/P2) retain anti-fibrotic activity, while offering practical advantages for clinical translation. Compared with freshly isolated cells (hAMSC/P0), expanded hAMSCs can be banked and quality-controlled and represent a more feasible product for clinical manufacturing in the context, for example, of ongoing clinical trials using MSCs to treat IPF (NCT05016817).

Intraperitoneal delivery (systemic exposure with reduced first-pass lung trapping vs. intravenous delivery) and the dose (4 × 10^6^ cells) used in this study were chosen on the basis of preclinical MSC-fibrosis literature, where doses of 10^5^ to 10^7^ cells and various delivery routes (IV, IP, IT) have been employed [67]. However, we are aware that dose-response studies and route optimization are needed for clinical translation.

The choice to study the progression of pulmonary fibrosis and the effect of cell-based treatment up to 14 days after bleomycin instillation might appear to represent a relatively short observation period, given that the progression of fibrosis can vary over time and treatment effects may not be sustained. In our previous experiments, which were performed up to day 28 [45], the fibrotic phenotype remained stable after day 14 and the treatment effect persisted, supporting day 14 as a suitable endpoint for evaluating anti-fibrotic efficacy. While longer follow-up would be useful to assess durability, it is unlikely to alter the overall conclusion that the intervention reduces established fibrosis.

Overall, our findings offer promising prospects for the development of novel cell-based therapies for lung fibrosis, emphasizing the importance of timing in hAMSC administration to achieve synergistic actions on different macrophage populations to optimize the therapeutic outcomes. While this study focuses on investigating the possible association between the anti-fibrotic benefit of hAMSCs and coordinated effects on multiple macrophage populations, we acknowledge that other fibrotic pathways -such as TGF-β/Smad signaling, epithelial-mesenchymal transition, and extracellular matrix remodeling- may also contribute to the observed anti-fibrotic effects. Moreover, the present study did not identify the specific hAMSC-derived factors responsible for these effects. Our previous analysis of the miRNA cargo of extracellular vesicles (EVs) derived from the hAMSC secretome [68] identified a profile consistent with the promotion of macrophage polarization toward a regulatory phenotype. Further studies are therefore needed to determine whether hAMSCs directly or indirectly modulate these fibrotic pathways and to define the specific contribution of EVs to their anti-inflammatory and anti-fibrotic activity.

While our preclinical data demonstrate promising efficacy of early- and late-administered hAMSCs in a bleomycin-induced lung injury model, several translational challenges must be addressed before clinical application. In our study, cells were derived from a limited number of donors under controlled laboratory conditions and validated for their viability and phenotype. However, like other MSCs, hAMSCs can exhibit intrinsic donor-to-donor heterogeneity in age, sex and healthy status, which can affect their functional features, potentially influencing therapeutic consistency across batches. Future work will need to systematically evaluate inter-donor variability and standardized potency assays to identify robust, clinically relevant biomarkers of hAMSC function. Translating hAMSC therapy to the clinic will also require manufacturing processes under Good Manufacturing Practice (GMP) conditions that ensure batch-to-batch consistency in terms of cell identity, cell purity and cell function. Moreover, although MSCs are generally considered immunoprivileged, repeated administration can trigger alloimmune responses, necessitating long-term multi-dose preclinical monitoring before human trials. Similarly, although MSCs are considered a clinically safe treatment, future investigations should include surveillance for late-onset complications in relevant large-animal models to establish a favorable long-term safety profile for regulatory approval and clinical adoption.

In light of these considerations, our findings should be viewed as a proof-of-concept that supports further optimization and validation of hAMSC-based therapies. Further research in this area holds the potential to revolutionize the management of fibrotic conditions and improve the development of effective therapy for individuals with pulmonary fibrosis.

## Data Availability

The datasets generated for this study are available on request to the corresponding author.

## Supplementary Materials

The following supporting information can be downloaded at: https://www.mdpi.com/article/10.3390/pharmaceutics18081039/s1, Figure S1: hAMSCs promote weight gain in bleomycin-challenged mice whether administered early or late; Figure S2: Co-expression of iNOS and Arg1 in lung macrophages of bleomycin-challenged mice.

## Abbreviations

The following abbreviations are used in this manuscript:

hAMSC	Human Amniotic Mesenchymal Stromal Cell
EV	Extracellular Vesicles
Treg	T regulatory cell
ECM	Extracellular Matrix
iNOS	Inducible Nitric oxide Synthase
Arg1	Arginase 1
SPARC	Secreted Protein Acidic and Rich in Cysteine
CD169	Sialoadhesin
NAMs	Nerve- and Airway-associated Macrophages
IL	Interleukin
CCL	C-C motif Chemokine Ligand
CCR	C-C Chemokine Receptor
CD	Cluster of Differentiation
PBS	Phosphate Buffered Saline
Bleo	Bleomycin
HE	Hematoxylin and Eosin
α-SMA	α Smooth Muscle Actin
DAPI	4′,6-diamidino-2-phenylindole
BCIP/NBT	5-Bromo-4-Chloro-3-Indolyl-Phosphate/Nitro Blue Tetrazolium
HRP	Horseradish Peroxidase
AP	Alkaline phosphatase
DAB	Diaminobenzidine
CX3CR1	C-X3-C motif chemokine receptor 1
FOXP3	Forkhead box P3
