# Supplementary material for: Amniotic Mesenchymal Stromal Cell Administration Prevents and Stops Lung Fibrosis and Is Associated with Distinct Macrophage Signatures
Source: Pharmaceutics. 2026 Aug 20;18(8):1039. doi: 10.3390/pharmaceutics18081039 (PMC13516232; doi:10.3390/pharmaceutics18081039)
Supplement: Supplementary file 1 [file pharmaceutics-18-01039-s001.zip › pharmaceutics-4408808-supplementary.pdf]

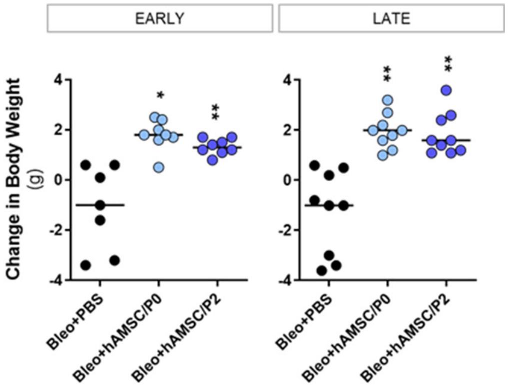


**Figure S1**. hAMSCs promote weight gain in bleomycin-challenged mice whether administered early or late. Changes in mouse body weight, in terms of difference between weight at sacrifice and weight before bleomycin challenge, with respect to different treatments (EARLY = treatments concomitant with bleomycin challenge; LATE = treatments at 7 days post-bleomycin challenge). Data are reported as scatter dot-plots with median, where each dot represents 1 mouse (n = 7–9 per experimental group). Between-group comparisons were performed with Kruskal-Wallis' test corrected for Dunn's multiple comparisons test. Statistical significance is indicated as *p<0.05; **p<0.01 vs control (Bleo+PBS) group.

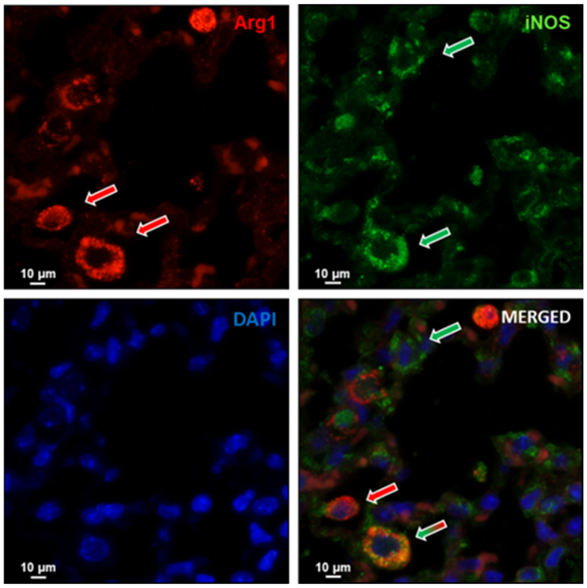


**Figure S2**. Co-expression of iNOS and Arg1 in lung macrophages of bleomycin challenged mice. Representative photomicrographs of lung sections from a bleomycin-challenged mouse (Bleo+PBS group) with immunofluorescence staining to detect the expression of Arg1 (red fluorescence), iNOS (green fluorescence), and Arg1/iNOS co-expression. DAPI (4',6-diamidino-2-phenylindole) is used for nuclear staining.
